# Practitioner review: health anxiety in children and young people in the context of the COVID-19 pandemic

**DOI:** 10.1017/S1352465820000636

**Published:** 2020-08-24

**Authors:** A. Haig-Ferguson, K. Cooper, E. Cartwright, M.E. Loades, J. Daniels

**Affiliations:** 1 Department of Psychology, University of Bath, Bath, UK; 2 Oxford Health NHS Foundation Trust, Oxford, UK; 3 Bristol Medical School, University of Bristol, Bristol, UK

**Keywords:** adolescent, child, COVID-19 pandemic, health anxiety, review

## Abstract

Health-related fear is a normal and common response in the face of the global pandemic of COVID-19. Children and young people are frequently being exposed to messages about the threat to health, including from the media and authorities. Whilst for most, their anxiety will be proportionate to the threat, for some, existing pre-occupation with physical symptoms and illness will become more problematic. There is a growing body of evidence that health anxiety may occur in childhood, however much of the literature is taken from research using adult samples. This practitioner review aims to give an overview of the assessment and treatment of health-related worries in children and young people in the context of the COVID-19 pandemic. This review is based on the limited existing evidence in this population and the more substantial evidence base for treating health anxiety in adults. We consider the adaptations needed to ensure such interventions are developmentally appropriate.

## Introduction

Worries about health are a common human experience thought to fall along a continuum in the general population (Salkovskis and Warwick, 2001). At the upper end of the continuum, individuals experience an obsessive fear of illness (Bilani *et al*., 2019) which may fall within the realm of health anxiety. Health anxiety as a diagnostic entity is characterised by a pre-occupation with either having or developing a serious physical illness, which is maintained by behaviours which are designed to ameliorate distress, but serve to inadvertently increase or maintain physical symptoms and anxiety. Recently changed to ‘illness anxiety disorder’ in the *Diagnostic Statistical Manual* [*DSM-5*; American Psychiatric Association (APA), 2013], the term health anxiety (HA) is still in common use by mental health professionals as this also refers to the evidence-based cognitive behavioural model of HA that is used in research and clinical settings (Salkovskis *et al*., 2003; Warwick and Salkovskis, 1990). In this paper, we seek to extrapolate evidence from the HA literature to inform how health-related worries in children and young people may be managed in practice, and more specifically, how this might relate to infectious disease pandemics such as COVID-19.

## Health anxiety in children and young people

HA as a disorder typically presents in early or middle adulthood (APA, 2013; Rask *et al*., 2016). There is some evidence to suggest that HA does occur in childhood and adolescence (Eminson *et al*., 1996; Oliver *et al*., 2018; Rask *et al*., 2012; Rask *et al*., 2016; Sirri *et al*., 2015; van Geelen *et al*., 2015; Wright and Asmundson, 2003), yet studies examining the prevalence of HA in children and young people suggest that few meet the full diagnostic criteria (Rask *et al*., 2016). This may be partly due to a lack of tailored, developmentally appropriate descriptions of how HA presents in children and young people (Fritz *et al*., 1997; Rask *et al*., 2016).

The *DSM-5* diagnostic criteria for illness anxiety disorder (previously known as HA) specifies that the following symptoms need to be present for at least 6 months: pre-occupation with illness, absence of somatic symptoms, hypervigilance of own health, self-monitoring for signs of illness, complete avoidance of medical care or conversely frequent seeking of medical care (APA, 2013). There are likely to be key differences in the presentation of HA in children and young people which relate to stages of development, particularly in relation to safety-seeking behaviours and ability to articulate what has precipitated the behaviour. For example, the pre-occupation with already having or contracting an illness may manifest as more generic concerns about health as detailed knowledge of disease and disease processes may be absent, especially in younger children. Like adults, children may display checking behaviours and seek reassurance directly from parents, while adolescents may be more prone to seeking reassurance more tacitly from friends or by searching for information online. Avoidance is also likely to be present, but the ability to articulate the root of the avoidance is likely to vary according to developmental abilities.

Parental reassurance, acquiescence and inadvertent collusion with safety-seeking behaviours will serve to maintain anxiety through prevention of disconfirmation and the associated physiological and emotional impact of safety-seeking behaviours we see in adults. Retrospective studies of adults with HA indicate that key symptoms, such as fears about becoming unwell, were present in childhood (Fink *et al*., 2004; Noyes *et al*., 2002), suggesting that the hallmark symptom of HA diagnosis, feeling pre-occupied by the idea that one might be ill, is present from an early age; we know less about the behavioural maintenance at this early age, however we may apply the mechanisms we see in other anxiety disorders.

Health-related worries and behaviours are common in children and young people (Rask *et al*., 2012; Rask *et al*., 2016; Sirri *et al*., 2015; Wright and Asmundson, 2003), with 15.7% of a sample of 14- to 19-year-olds reporting ‘clinically significant hypochondriacal symptoms’ (Sirri *et al*., 2015) and the parents of 2.7% of 5- to 7-year-olds reporting ‘considerable’ health anxiety symptoms in their children (Rask *et al*., 2012). Thus, even though prevalence of HA as a recognised disorder may not have been established, health-related worries appear to be relatively common.

HA has a significant impact on functioning in children and young people; health-related anxiety symptoms have been found to be significantly positively associated with mental health problems (including emotional disorders, distress and somatisation) across a range of age groups (Rask *et al*., 2012; Rask *et al.*, 2016; Sirri *et al.*, 2015). Additionally, younger children with higher levels of HA-related symptoms are more likely to present with functional somatic health problems than their peers (Rask *et al*., 2012; Rask *et al*., 2016). There are also economic costs. The medical costs for children with the highest levels of HA-related symptoms have been found to be 150 Euros more than those with the lowest levels of HA symptoms over a 2-year period, even when controlling for physical health conditions (Rask *et al*., 2016).

## Health related worries in the context of COVID-19

COVID-19 is an infectious disease caused by a novel coronavirus, SARS-CoV-2 [World Health Organization (WHO), 2020a] which, in most cases, results in mild symptoms such as a dry cough and fever. In a minority of cases, SARS-CoV-2 can lead to more severe complications (including acute respiratory distress syndrome) and can be fatal (Sohrabi *et al*., 2020). Children and young people are reported to be asymptomatic or relatively more mildly affected with few severe cases reported (Lu *et al*., 2020).

Since the global spread of COVID-19, and the declaration of a pandemic by the World Health Organization on 11 March 2020 (WHO, 2020b), the worldwide health context is far from typical. There is a genuine and unprecedented threat to health and survival, which results in health concern. This threat is compounded by media focus on numbers of positive cases and deaths (Taylor, 2019) rather than negative cases and recoveries. This is likely to increase public perception of risk of contracting the virus, as well as over-estimation of risk if they do (Wheaton *et al*., 2012).

In the face of an unprecedented infectious disease pandemic, it is natural to feel anxious and to be concerned about one’s own health and the health of others. For most, anxiety will be a proportionate and adaptive response to the threat posed by the virus, motivating individuals to engage in sensible, precautionary health behaviours (Taylor, 2019). However, for a minority, particularly those who may be pre-disposed to anxiety and/or who lack coping resources, COVID-19 might act as a ‘critical incident’ that triggers health-related worries. In these individuals, ‘normal’ health anxiety escalates and becomes distressing and debilitating; while for others, health-related fears are transitory and simmer down once the threat begins to pass. It is the former that we focus on in this review.

## Health-related worries in children and young people and COVID-19

There are both individual and systematic factors that may render children and young people particularly vulnerable to health-related worries in the context of COVID-19. It is important to acknowledge that there are key differences in the presentation of anxiety between children and adolescents (Waite and Creswell, 2014), such as the expression of affect through physical sensations in younger children. We distinguish between these two groups where possible and where there is an evidence base to support such differentiation.

### Individual factors – cognitive and developmental

For children and young people, COVID-19 will probably be the first major global health threat they will have been exposed to. This may shape or disrupt the formation of their emergent beliefs about the world being a ‘safe place’ with regard to health. There are a multitude of messages about the omnipresent threat: significant changes to behaviour have been enforced, including school closures as disease containment measures, alarming public health messages about staying home, avoiding people and keeping ‘safe’ being repeatedly communicated through various media.

Children’s cognitive ability to process and understand complex information is more limited and concrete in comparison with adults (Inhelder and Piaget, 1958). This makes it more difficult for children to understand abstract information, such as a ‘viral’ health-related threat. Their emergent beliefs are also more malleable than those of adults and are influenced and shaped by significant others (Cantor *et al*., 2018). Over the course of adolescence, the individual’s own ideas and beliefs develop, often influenced by those of peers. Their cognitive abilities develop so that they can make sense of the world and events in more abstract, complex ways, and can hold several possibilities in mind. Adolescents are also increasingly able to take ownership over health-relevant behaviours (Turner-Cobb, 2013); however, this and earlier childhood is a sensitive time in terms of their formative development of their understanding of the world.

### Individual factors – pre-existing mental health or physical health problems

Some children and young people are more vulnerable to developing health-related worries in this context due to pre-existing anxiety and/or experiences. In university students, prior anxiety sensitivity and obsessive-compulsive symptoms and beliefs increased the risk of experiencing pandemic-related anxiety in the context of swine flu (Brand *et al*., 2013). This may also be relevant for children and young people who have pre-existing anxiety and a heightened sense of the world as a ‘dangerous’ place prior to COVID-19. Those who experience the death or serious illness of someone in their personal or social network as a result of the disease, and those who live with parents who are particularly worried about the virus and its impact, may be more vulnerable to developing health-related worries.

Some children and young people have underlying physical health problems which render them more vulnerable to respiratory-type illnesses like COVID-19, including those with asthma and the immunosuppressed. These individuals will need to take a higher level of precautionary measures to keep ‘safe’; it is these additional measures and higher sense of threat of illness that leave them at higher risk of ongoing health-related worries. Due to lack of complex cognitive abilities at a young age, children may generalise the perception of threat.

### Systemic factors – parent and carer mental health

Children’s beliefs about illness and their responses to symptoms are shaped by those of significant others, particularly their primary caregivers, most typically their parents (Turner-Cobb, 2013). There is evidence of an association between parent and child HA symptoms (Koteles *et al*., 2015; Marshall *et al*., 2007; Remmerswaal and Muris, 2011; Wright *et al*., 2017). This may arise in several ways. Firstly, a significant proportion of learning takes place, particularly at a young age, through observation and imitation of significant others. Secondly, the responses of those significant others reinforce particular behaviours that the child displays (Bandura, 1977). Thus, interacting with others is a key social-cognitive process that inevitably shapes the child’s belief system.

Emerging evidence suggests that anxiety levels in adults in the general population have increased since ‘lockdown’ (Bentall, 2020), with HA in the adult population estimated to have increased by around 10% (Rettie and Daniels, 2020). The psychological impact of ‘lockdown’ for adults is likely to be profound and long lasting for many (Brooks *et al*., 2020). Adults are likely to be experiencing stress and general pressures associated with lack of access to normal activities of daily living, and financial concerns due to the economic consequences of lockdown (Brooks *et al*., 2020). In addition, many parents have the pressure of home-schooling their children whilst also working themselves.

When parental mental health is compromised, there is undoubtedly some degree of impact on the child. This may be directly through modelling of anxious behaviour or reduced capacity for the parent to support the child with their own anxiety, for example. Indeed, there is a large body of evidence to support the notion of intergenerational transmission of anxiety (Lawrence *et al*., 2019). This is likely to be further compounded by the curtailed access to both adult and child mental health support, and to other supportive adults such as educational professionals during school closures.

## Assessment

There is a high degree of co-morbidity in anxiety disorders (Creswell *et al*., 2014; Kendall *et al*., 2010; Leyfer *et al*., 2013) and mood disorders (Essau, 2003) across the age range. However, research indicates it is possible to separate diagnostic features of different forms of anxiety reliably (Spence, 2017). Even though anxiety disorders share transdiagnostic processes, they can be separated by their primary focal point of anxiety (Ferdinand *et al*., 2007), therefore accurate and thorough assessment is imperative to access the right support.

In children and young people, a multi-method (including interviews, questionnaires and observational approaches) and multi-informant (self-, parent- and possibly teacher-report) approach to assessment is recommended (Hudson *et al*., 2014; Kazdin, 2003; Silverman and Ollendick, 2005).

Structured diagnostic interviews are generally considered to be the gold standard assessment to identify mental health disorders in children and young people (Spence, 2017). These tend to seek information from multiple informants, most often the affected child or young person and a parent. The most used measures include the Anxiety Disorders Interview Schedule (Silverman and Albano, 1996) and the Kiddie Schedule of Affective Disorders and Schizophrenia (Kaufman *et al*., 1997), both of which include questions pertaining to health-related worries, although sections pertaining to a diagnosis of health anxiety are comparatively brief.

Self-report questionnaires can be useful as a screening tool to complement a clinical interview; however, there are no specific questionnaires or subscales on generic questionnaires which measure HA in children and young people. A version of the Illness Attitudes Scale (IAS; Kellner, 1986) has been developed for children, although its psychometric properties are yet to be established. Validated and reliable self-report measures developed for use in adults may be useful (see Table 1), although their psychometric properties in children and young people are unknown. There are also several questionnaires that have been developed for research purposes to examine parental report of children’s health-related worries; however, these have not been extensively used in clinical practice (Rask, 2019). These include the parent version of the Illness Worry Scale (IWS-p; Garralda and Rangel, 2001), Fetal Health Anxiety Inventory (Reiser and Wright, 2019) and the Protect subscale of the Adult Responses to Children’s Symptoms (ARCS; Van Slyke and Walker, 2006).

**Table 1.** Self-report measures of health anxiety developed for use with adult populations

	Health Anxiety Inventory (HAI) (Salkovskis *et al*., 2002)	Illness Attitude Scales (IAS) (Kellner, 1986)	Whiteley Index (Pilowsky, 1967)
Description	64 items	29 items	14 items
	Each item rated 0–3	Each item rated 0–4	Dichotomous scoring (yes/no)
		Nine subscales: worry about illness; concerns about pain; health habits; hypochondriacal beliefs; thanatophobia (fear of death); disease phobia; bodily pre-occupations; treatment experience; effects of symptoms	
Total scale range	0–192	0–108 (only 27 items are used for the sum score)	0–14
Other versions	Short version (SHAI) – 18 items, total scale range of 0–54	CIAS is a version adapted for ages 8–15 (Wright and Anderson, 2003)	Short version – 7 item version (Fink *et al*., 1999)
Clinical cut-offs	HAI: ≥67 (Hedman *et al*., 2015); SHAI: ≥ 27 (Abramowitz *et al*., 2007)	IAS: ≥45–47 (Hiller *et al*., 2002; Hedman *et al*., 2015)	14 item: ≥5–8
			7 item: ≥2.5–5

Additionally, there are clinician rating scales for HA (see Table 2), although these also have not been developed specifically with children and young people in mind.

**Table 2.** Clinician-administered measures of health anxiety

	Rating scale of hypochondriacal beliefs (Kellner, 1986)	Heightened illness concern severity scale (Fallon, 2001)	The modified Yale–Brown Obsessive-Compulsive Scale for Hypochondriasis (H-YBOCS-M) (Skritskaya *et al*., 2012)
Description	1-item scale: from 1 (absent, no concerns about physical symptoms or illness) to 9 (extremely severe persistent and continuous beliefs he/she suffers from a physical illness; reassurance by a physician does not alter the belief)	1-item scale: from 1 (no heightened illness concern) to 7 (extreme heightened illness concern; amongst the most ill of all heightened concern patients, causing extreme distress and significant social and occupational impairment)	19 items looking at thoughts, behaviours and avoidance all typical of health anxiety
Psychometric properties	Good inter-rater reliability	Is sensitive to change (Fallon *et al*., 2003)	Adequate internal consistency and is sensitive to change. Moderately correlated with the Whiteley Index (Skritskaya *et al*., 2012)

## Formulation

The current understanding of HA is primarily informed by cognitive behavioural models (Abramowitz *et al*., 2002; Asmundson *et al*., 2004; Salkovskis *et al*., 2003; Taylor and Asmundson, 2004; Warwick and Salkovskis, 1990). Cognitive behavioural therapy (CBT) models have the strongest empirical underpinning and evidence base for the treatment of anxiety disorders (e.g. see Abramowitz *et al*., 2009; Marcus *et al*., 2007; Marcus and Norris, 2014; Rachman, 2012; Taylor and Asmundson, 2004). The Salkovskis *et al*. (2003) model of HA posits that through experience, health-related beliefs are formed that may lead to misappraisal of innocuous bodily states, then further pre-disposing the person to the development of HA. Once established, episodes of HA are usually triggered by incoming health-related stimuli, which are interpreted to be a relevant threat. This raises anxiety, precipitating use of safety-seeking behaviours to reduce anxiety and prevent feared health catastrophes; however, these behaviours prevent disconfirmation of beliefs and perpetuate this cycle of anxiety.

**Figure 1.**
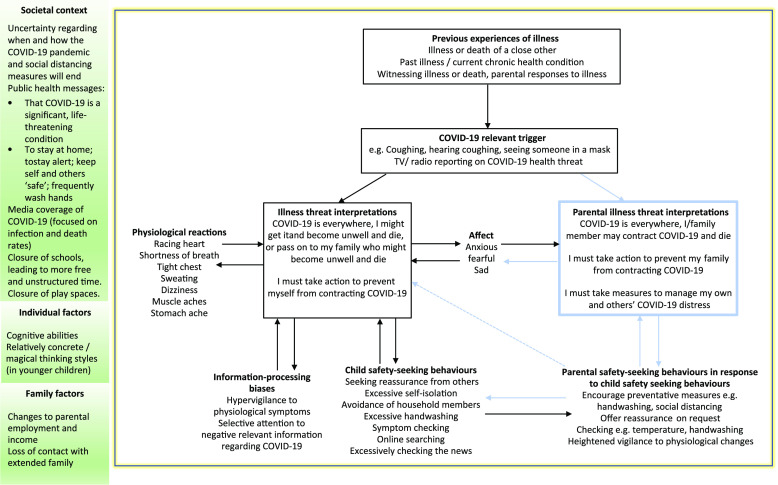
A simplified model of health anxiety as applied to children and young people and their families. Adapted from Salkovskis *et al*. (2003).

Figure 1 presents the empirically grounded CBT formulation for HA in adults (Salkovskis *et al*., 2003), adapted for children and young people in the context of COVID-19. This formulation outlines the interrelated cognitive, behavioural, affective and physiological factors in HA outlined above, and includes the role of parents in the maintenance of anxiety in children and young people (e.g. Murray *et al*., 2009), with further considerations to the wider societal context in which the child or young person lives.

The hypothetical formulation of health anxiety in children and young people presented here is drawn from the known characteristics of health anxiety in adults, and the empirically grounded evidence-based model of health anxiety in adults. It is therefore subject to the inherent limitations of extrapolating mechanisms that underpin anxiety in adults to children, and it is within this context that the model should be considered. Further research is needed to understand the underpinning mechanisms that maintain health anxiety in children and young people and the adaptations that may be required to accommodate particular developmental stages.

For children and young people, triggers for anxiety are likely to take the form of physical sensations (e.g. urge to cough/coughing) or health threat information (e.g. TV programmes, news updates). As children and young people are less likely to scrutinise the quality and relevance of incoming information, a threat response may be triggered at a lower threshold than adults. Cognitions might typically centre on the risk and likelihood of becoming ill with COVID-19, alongside thoughts of how catastrophic this could be (i.e. ultimately death of self/parent). Behaviours resulting from these health-related fears would be strategically aimed at reducing both distress and risk of contracting COVID-19, with the unintended consequence of increasing anxiety through prevention of disconfirmation, e.g. symptom checking, overly restrictive practices, excessive handwashing. This process also serves to increase the perception of worrying physical sensations, through symptom hypervigilance for example, or interoceptive experience of physiological arousal associated with anxiety. This further acts as a health-related stimulus, elevating anxiety and perceived health risk, and so the cycle continues. This is particularly problematic due to the limited ability that children may have to understand and express their own emotions, therefore the experience of emotions themselves may be perceived as threatening.

Within this adapted formulation we also present key mechanisms between the parent and child that may contribute to the maintenance of distress, albeit inadvertently. Illness beliefs in the anxious parent trigger safety-seeking strategies that are designed to keep both themselves, and the child, emotionally and physically safe. However, parental safety-seeking behaviours, and the anxieties that underpin them, are likely to raise salience of the health threat to the child. This in turn drives the child’s own safety-seeking behaviours, which also seek to reduce distress and threat to physical health. Parental safety-seeking behaviours are also likely to influence the child’s behaviours without an explicit cognitive route, e.g. parent repeatedly checks for signs of illness, this behaviour is repeated by the child, learned via modelling and reinforced by the absence of negative consequences. This, within a societal context of public health messages that everyone must stay home in order to be safe and keep others safe, and with schools and workplaces closed, may lead to intensifying difficult dynamics within the family home.

## Treatment

Specific interventions for HA in children and young people are lacking. Therefore, it may be useful to draw together existing adult treatments that address health-related anxiety in complex medical contexts (Daniels *et al*., 2020; Daniels and Sheils, 2017; Tyrer *et al*., 2017) and established knowledge on how psychological treatments are adapted for children and young people. While there is research exploring health-related fears during infectious disease outbreaks, this does not extend to treatment studies, which are primarily focused on trauma and generic anxiety and depression, so we have little new to learn in this respect. The evidence-based model commonly used for HA is described by Salkovskis *et al*. (2003); however, this requires adaptation for use with children and young people to ensure it is developmentally appropriate (Rask, 2019).

### The evidence for cognitive behavioural therapy

There is evidence to support the use of CBT for childhood anxiety, ranging from low-intensity interventions (bibliotherapy and E-therapy) to high-intensity interventions (nine to 20 face-to-face sessions: Creswell *et al*., 2014; Creswell *et al*., 2020; James *et al*., 2015). The response to CBT is dependent on the type of anxiety presentation; children and adolescents with social anxiety may experience a slower rate of change post-treatment than those suffering from generalised anxiety (Hudson *et al*., 2015). Studies have tended not to conduct separate trials for children and adolescents, despite different presentations in these two groups. Zhou *et al*. (2019) conducted a network meta-analysis of psychotherapies delivered within a range of conditions and modalities, e.g. individual and group, internet and face-to-face, with and without parental involvement. CBT treatments had the best outcomes in terms of quality of life and functioning. Schwartz *et al*. (2019) conducted a meta-analysis of anxiety interventions for children and found that 10 of the 11 CBT interventions evaluated were effective in reducing anxiety in children and young people. However, it is important to note that neither of these studies specifically focused on health-related worries.

CBT is effective in treating HA in adults (Cooper *et al*., 2017; Hart and Bjorgvinsson, 2010; Newby *et al*., 2018). A brief five to 10 sessions of adapted CBT for HA (known as CBT-HA) was effective in improving HA symptoms (Tyrer *et al*., 2014), improvements were sustained over 5 years and the intervention was cost-neutral (Tyrer *et al*., 2017). CBT has been found to be more effective than psychoeducation, clinical support and monitoring (Newby *et al.*, 2018), whilst cognitive and exposure-based approaches have also both been found to be effective (Weck *et al*., 2015).

We propose that formulation-driven CBT for health-related worries and HA is probably the best treatment available for children and young people with HA. This will be most effective if adapted to be developmentally appropriate. Creswell *et al*. (2020) suggest that disorder-specific treatments may improve outcomes for young people with anxiety, but that evidence-based models need to be developed and tested in large clinical trials. In this paper we therefore provide an adaptation of the adult disorder-specific model, with developmental considerations. This hypothetical model is underpinned by existing research into health-related fear and anxiety in children and young people and draws together what is known to work for children with anxiety and adults with health anxiety.

### What a CBT intervention for health-related worries with a child or young person could involve

The aim of CBT for HA is to reduce distress, reduce unhelpful safety-seeking behaviours and enable consideration of alternative explanations of physical sensations that are being misinterpreted as signs of disease (Daniels and Loades, 2017; Tyrer *et al.*, 2014).

As with adult HA interventions and good practice in CBT for children and young people (Fuggle *et al.*, 2012; Stallard, 2002, 2005), assessment and formulation is the necessary foundation of individualised treatment. Once a shared understanding is reached of how health-related worries manifest, including the cyclical nature and the role of safety-seeking behaviours, it is the latter which becomes the focus of the intervention. In the context of COVID-19, the intervention might also involve a focus on tolerance of uncertainty, and recognition that the excessive use of precautionary measures may be increasing rather than decreasing anxiety about contracting COVID-19. This will foster an understanding of what constitutes necessary precautionary measures which are no more, or no less than required (see Table 3). Adaptations must be made to ensure efficacy of treatment for children and young people. Rask (2019) suggests that duration and creative content of sessions (role play, using cartoons, drawing or using play) should be key considerations when adapting a CBT intervention for children, in addition to adjustments for cognitive and social developmental stages. This might include simplification of concepts, scaffolding knowledge and emotional literacy through increased psychoeducation about both illness and about emotions, including family members in treatment, and use of creative, fun activities to engage children and young people (Stallard, 2002). Some of the intervention strategies commonly used in CBT for HA are conceptually abstract and require cognitive reasoning, which would require adaptation to stage of cognitive emotional development.

**Table 3.** What CBT for health-related worries in children and young people in the COVID-19 context might involve (adapted from Tyrer *et al*., 2011)

Formulation of the young person’s worst fears related to COVID-19, and how this fits with family attitudes and behaviours during the pandemic, as well as during previous episodes of ill-health
Introduction to the possibility of alternative explanations of physical symptoms, e.g. anxiety about COVID-19 is causing significant distress and physiological arousal, rather than the young person being ill with COVID-19
Evaluation of risk of COVID-19, acknowledging the lack of certainty in the context of this novel coronavirus
Consideration of the negative consequences of health anxiety for the young person and their family
Exploration of the hypothesis that fear of COVID-19 is having more of a negative impact on the young person than COVID-19 itself
Identification of residual physical sensations related to anxiety and attentional bias for these symptoms
Reduction and testing of safety-seeking behaviours that may be helping to maintain health anxiety, e.g. excessive bodily checking (young person) and providing excessive reassurance (parents)
Building resilience in the family to manage COVID-19 difficulties, e.g. strategies to facilitate home-schooling and maintain a sense of routine, considerations for parents working from home, maintaining healthy habits, e.g. sleep hygiene, healthy eating, exercise

### How parents might be involved in treatment

Rask (2019) outlines three fundamental differences between child HA and adult HA, which point towards a central role of parents/caregivers in the treatment of HA and health-related worries in children:

1. There is a qualitative difference to a child’s thinking processes compared with an adult, and they will have a different understanding of illness and disease.
2. Reassurance seeking will often be directed to adults in the child’s life such as parents, caregivers and teachers rather than health professionals.
3. Maintaining factors will be inextricably linked to the beliefs and psychosocial functioning of parents.

While there are differences between HA in adults and in those aged under 18, it is also important to consider that there are also likely to be developmental differences between children and young people (Waite and Creswell, 2014). For example, the child’s age and developmental stage may influence decision-making and relevance of parental involvement in therapy. This may be helpful for younger children but may actually feel disempowering and inhibit engagement for adolescents. It may be that involving parents in some but not all sessions, or at the beginning or end of sessions only could be useful in this circumstance. Evidence regarding parental involvement in children’s anxiety treatment is mixed. Two recent meta-analyses included studies reflecting a wide range of ages (3–18 years) and concluded that parental involvement has little or no impact on outcome (Thulin *et al*., 2014; Zhou *et al*., 2019), whilst other studies report that for children aged 4–12 years, parent-led CBT is effective in treating anxiety (Cartwright-Hatton et al., 2011; Creswell *et al*., 2017; Waters *et al*., 2009). Parent-led treatment for HA in children could therefore be a useful approach and warrants further exploration.

Creswell and colleagues (2020) suggest that parental involvement is not binary; it is the quality of involvement that is important, recommending a nuanced approach to involving parents, which may be dependent on the individual formulation for that child. There is evidence that involvement of parents in online CBT programmes produces significantly larger effect sizes than programmes that do not involve parents (Grist *et al*., 2018). There is also evidence that if parental involvement in low-intensity therapy such as bibliotherapy is closely supported by a therapist, outcomes are enhanced (Cobham, 2012; Lyneham and Rapee, 2006; Thirlwall *et al*., 2013).

### Delivering psychological therapies in the COVID-19 context

Dalton *et al*. (2020) indicate that it is of utmost importance to talk about the pandemic with children and young people in a developmentally appropriate manner. This is particularly the case if children and young people experience distress related to COVID-19. In the context of lockdown measures, social isolation and physical distancing, face-to-face therapy is not practical or safe, therefore it is important to consider alternative methods in delivering treatment. There is a body of evidence indicating that computerised CBT is effective in child, young people and adult populations (Grist *et al*., 2018; Hollis *et al*., 2017; Newby *et al*., 2020; Olthuis *et al*., 2016; Thew, 2020; Vigerland *et al*., 2016). Evidence also suggests that transdiagnostic online CBT for anxiety in children and young people can be as effective in emergency contexts: in 2012, after a series of violent earthquakes in New Zealand, an online therapist-assisted CBT package was found to be effective at reducing anxiety (Moor *et al*., 2019). Moreover, many therapists are now delivering CBT via video platforms during the pandemic out of necessity. In response to these and other changes, the British Psychological Society (BPS) (2020) have produced guidance on adaptation for this mode of delivery which recommends working creatively with children on a video call by utilising screen sharing; managing expectations through having a set session format; ensuring privacy; and being alert to signs of distress and having a plan as to how to respond to this remotely. The BPS guidance is also clear that therapy delivered via video may not be appropriate for all children and young people.

In the absence of online HA-specific interventions for children and young people, adapting existing online evidence-based programmes for anxiety to address health-related worries relevant to COVID-19 specifically may hold promise. The adaptations needed would include specific content about coronaviruses and COVID-19, and about concerns children and young people may have about the health threat to adults as well as to themselves. This would need to acknowledge the ongoing uncertainty about COVID-19 and to include a focus on coping with this uncertainty, as well as uncertainty about how long disease containment measures will be in place and how they will be lifted. Content and techniques, including practical exercises, would need to consider physical distancing and disease containment measures. Specific suggestions about how to seek social support and to maintain social contact within these limitations may also be helpful. Further research is needed to establish if existing transdiagnostic programmes, delivered online, are effective in the pandemic context at reducing health-related worries.

## Conclusion

In the context of a global pandemic, some degree of health-related fear is normal and adaptive. However, for a minority of children and young people, this health-related fear may become particularly distressing. It may interfere substantially with their functioning and persist over time, in a way that we recognise in HA. For these children and young people, adopting a multi-informant approach to assessment, including using existing HA scales to complement clinical interview, could help to establish the extent of their HA. An individualised formulation of the problem, including the cognitive, behavioural and emotional components, based on the CBT model, should be developed. Consideration of developmental factors such as cognitive ability, emotional literacy, intergenerational transmission of beliefs and parental modelling or reinforcement of behaviours, is an important part of the formulation. Adapting existing evidence-based HA treatments for adults and offering these to children and young people may be most helpful. More research is needed to establish age-appropriate diagnostic criteria and standardised tools for assessment of HA in younger children, and to evaluate developmentally appropriate treatment intervention/programmes.

## Key practitioner messages

1. Raised health-related worries during the COVID-19 pandemic are normal and do not necessarily require treatment.
2. For some, the level of anxiety experienced during the pandemic may be understood in terms of HA, which is treatable using cognitive behavioural models.
3. Although the majority of research into HA focuses on adults, evidence suggests that children and young people also develop health-related worries and HA, and may be particularly susceptible during global health crises.
4. When treating HA in children and young people, it is important to make development adaptations and consider involvement of parents in treatment.
5. Given the current government mandated measures to manage the spread of COVID-19, alternatives to face-to-face treatment should be considered, such as internet-delivered CBT.
